# Freestanding graphene/MnO_2_ cathodes for Li-ion batteries

**DOI:** 10.3762/bjnano.8.193

**Published:** 2017-09-14

**Authors:** Şeyma Özcan, Aslıhan Güler, Tugrul Cetinkaya, Mehmet O Guler, Hatem Akbulut

**Affiliations:** 1 Sakarya University, Engineering Faculty, Dept. of Metallurgical & Materials Engineering, Esentepe Campus, 54187, Sakarya, Turkey

**Keywords:** CR2016 coin cells, freestanding cathode, graphene, Li-ion battery, MnO_2_

## Abstract

Different polymorphs of MnO_2_ (α-, β-, and γ-) were produced by microwave hydrothermal synthesis, and graphene oxide (GO) nanosheets were prepared by oxidation of graphite using a modified Hummers’ method. Freestanding graphene/MnO_2_ cathodes were manufactured through a vacuum filtration process. The structure of the graphene/MnO_2_ nanocomposites was characterized using X-ray diffraction (XRD) and Raman spectroscopy. The surface and cross-sectional morphologies of freestanding cathodes were investigated by scanning electron microcopy (SEM). The charge–discharge profile of the cathodes was tested between 1.5 V and 4.5 V at a constant current of 0.1 mA cm^−2^ using CR2016 coin cells. The initial specific capacity of graphene/α-, β-, and γ-MnO_2_ freestanding cathodes was found to be 321 mAhg^−1^, 198 mAhg^−1^, and 251 mAhg^−1^, respectively. Finally, the graphene/α-MnO_2_ cathode displayed the best cycling performance due to the low charge transfer resistance and higher electrochemical reaction behavior. Graphene/α-MnO_2_ freestanding cathodes exhibited a specific capacity of 229 mAhg^−1^ after 200 cycles with 72% capacity retention.

## Introduction

Nowadays low cost, clean and sustainable energy storage requirements with high performance are of great need because of rapid improvement of mobile and stationary electronic applications. Lithium-ion batteries have been one of the key energy storage devices to meet these energy demands since the last century [1]. However, increased capacity and energy density of Li-ion batteries is desired in order to store more, efficient energy. Although researchers have made significant progress in the development of high capacity anode electrodes, such as SnO_2_ [2], Sn-Ni [3], and Si [4], the performance of cathodes has been bottlenecked by the energy density and capacity of Li-ion batteries. In commercial Li-ion batteries, LiCoO_2_, which has a specific capacity of 140 mAh/g, is used as the cathode material although it has many disadvantages such as high cost, toxicity and limited sources. Therefore, researchers have been developing different cathode materials such as LiMn_2_O_4_ and LiFePO_4_, which have a capacity of merely 150 mAh/g and 170 mAh/g, respectively [5–6].

Manganese dioxide (MnO_2_) is one of the most promising metal oxide as a replacement for the Li-ion electrode material owing to its high theoretical capacity (308 mAh/g), environmental friendliness and low cost [7]. It has gained a great deal of attention because of its wide application in areaa such as catalysts for Li–air batteries [8], molecular sieves [9] and electrodes in rechargeable batteries [10–12]. However, its drastic volume change, aggregation problems, and poor electronic conductance (resulting in low cyclability) has extremely limited its potential applications [10,13]. Therefore, nanostructured MnO_2_ has been fabricated and used with carbon materials to achieve excellent conductivity with a large specific surface area [14]. On one hand, reducing the dimensions of the electrode particles from the micrometer to the nanometer regime can enhance the ion exchange rate in Li-ion batteries [15], while on the other hand, supporting the cathode with carbon materials such as carbon nanotubes, acetylene black and graphene, helps to improve the conductivity of the electrode. Among these carbon materials graphene has become one the most attractive carbon support materials with its extraordinary properties.

Graphene is a two-dimensional (2D) atomic-scale honeycomb lattice made of carbon atoms. Its unique properties such as high electrical and thermal conductivity, high chemical stability, large specific surface area and high mechanical strength have made graphene one of the most promising materials for energy storage devices [16]. In recent reports, MnO_2_/graphene composite electrodes have been used in order to enhance the electrical conductivity and prevent volume change during charge–discharge cycles [17].

MnO_2_ has many crystallographic polymorphs including α-, β-, δ-, γ-, ε-, and λ-MnO_2_. The electrochemical characteristics of MnO_2_, such as electrocatalytic activity, specific capacity and oxygen reduction reaction, vary according to its crystalline structure and morphology [18]. However, there are few reports explaining their electrochemical reaction response relating to their different manganese oxide crystalline structures.

In this work, different polymorphs of MnO_2_ (α-, β-, and γ-) were produced by a microwave hydrothermal method. Freestanding graphene/MnO_2_ cathodes were manufactured through a vacuum filtration process without using any additional additives such as a binder. The effect of the different polymorphs, α-, β-, and γ-MnO_2_, on the structural and electrochemical properties of the manufactured, freestanding graphene/MnO_2_ cathodes was investigated. To the best of our knowledge, this study is the first to investigate the electrochemical performance of freestanding graphene/MnO_2_ cathodes for Li-ion batteries. The freestanding graphene/MnO_2_ cathodes exhibit a high specific capacity and excellent electrochemical cycling performance.

## Experimental

### Preparation of MnO_2_ phases

The α-, β-, and γ-MnO_2_ phases were synthesized by a microwave-assisted hydrothermal method. α-MnO_2_ nanowires and β-MnO_2_ nanorods were prepared according to our previous report [8]. To prepare γ-MnO_2_, 1.83 mg of (NH_4_)_2_S_2_O_8_, 1.35 mg of MnSO_4_ and 3 mmol were dissolved in 80 mL of distilled water. Then, the resulting solution was transferred into a Teflon (PTFE)-lined autoclave, sealed and placed in a microwave oven (Milestone ROTOSYNTH). The hydrothermal reaction was carried out for 60 min at 90 °C. Then the autoclave was cooled down to room temperature and the as-prepared black precipitate was filtered and washed several times with distilled water. γ-MnO_2_ was obtained after drying at 80 °C in a vacuum oven for 12 h.

### Preparation of freestanding graphene/MnO_2_ electrodes

Graphite oxide (GO) was synthesized according to a modified Hummers’ method [19] by using pretreated graphite flakes as the starting material, as explained in a previous study [17]. The freestanding graphene/MnO_2_ cathodes were prepared via a vacuum filtration technique. Firstly, 30 mg of GO was dissolved in 50 mL distilled water by ultrasonication for 1 h to obtain GO. Then 30 mg of as-synthesized MnO_2_ was added to the solution and ultrasonicated for another 1 h. The GO/MnO_2_ solution was filtered on a PVDF membrane by a vacuum filtration technique. In order to reduce the GO to graphene, the as-synthesized GO/MnO_2_ was subjected to a hydrazine solution after filtration of GO. 5.6 mL of a hydrazine solution was slowly poured onto GO/MnO_2_ and filtered. Then the obtained solid was peeled-off from the PVDF membrane and the freestanding graphene/MnO_2_ was obtained (approximate thickness is 15 μm). This process was carried out for all MnO_2_ phases.

The microstructural morphology of the freestanding graphene/α-, β-, and γ-MnO_2_ composite cathodes was characterized using scanning electron microscopy (SEM). The structural and phase investigation of the freestanding cathodes was tested using X-ray diffraction (XRD) and Raman spectroscopy.

### Electrochemical characterization of graphene/MnO_2_ cathodes

A CR2016 coin cell was used to investigate the electrochemical performance of the produced freestanding composite cathodes, assembled in an Ar-filled glove box. In this coin cell, the produced cathodes were used as a working electrode, and lithium foil was used as an anode. 1 M lithium hexafluorophosphate (LiPF_6_) was dissolved in ethylene carbonate (EC) and dimethyl carbonate (DMC) (EC/DMC, 1:1 v/v), which was used as the electrolyte. In order to separate the electrodes, a microporous polypropylene membrane was used. Electrochemical tests of the cathodes were implemented between 1.5 and 4.5 V at a constant current density of 0.1 mA cm^−2^. The specific capacity of the freestanding graphene/MnO_2_ cathodes was calculated depending on the active mass of the graphene/MnO_2_ composite (about 20 mg) on Al foil. The resistance of the electrodes was evaluated via electrochemical impedance spectroscopy (EIS) using a Nyquist curve in the frequency range 1000 kHz–0.1 Hz with an AC amplitude of 10 mV with fresh electrode applied before the electrochemical cycling test.

## Results and Discussion

**Figure 1:**
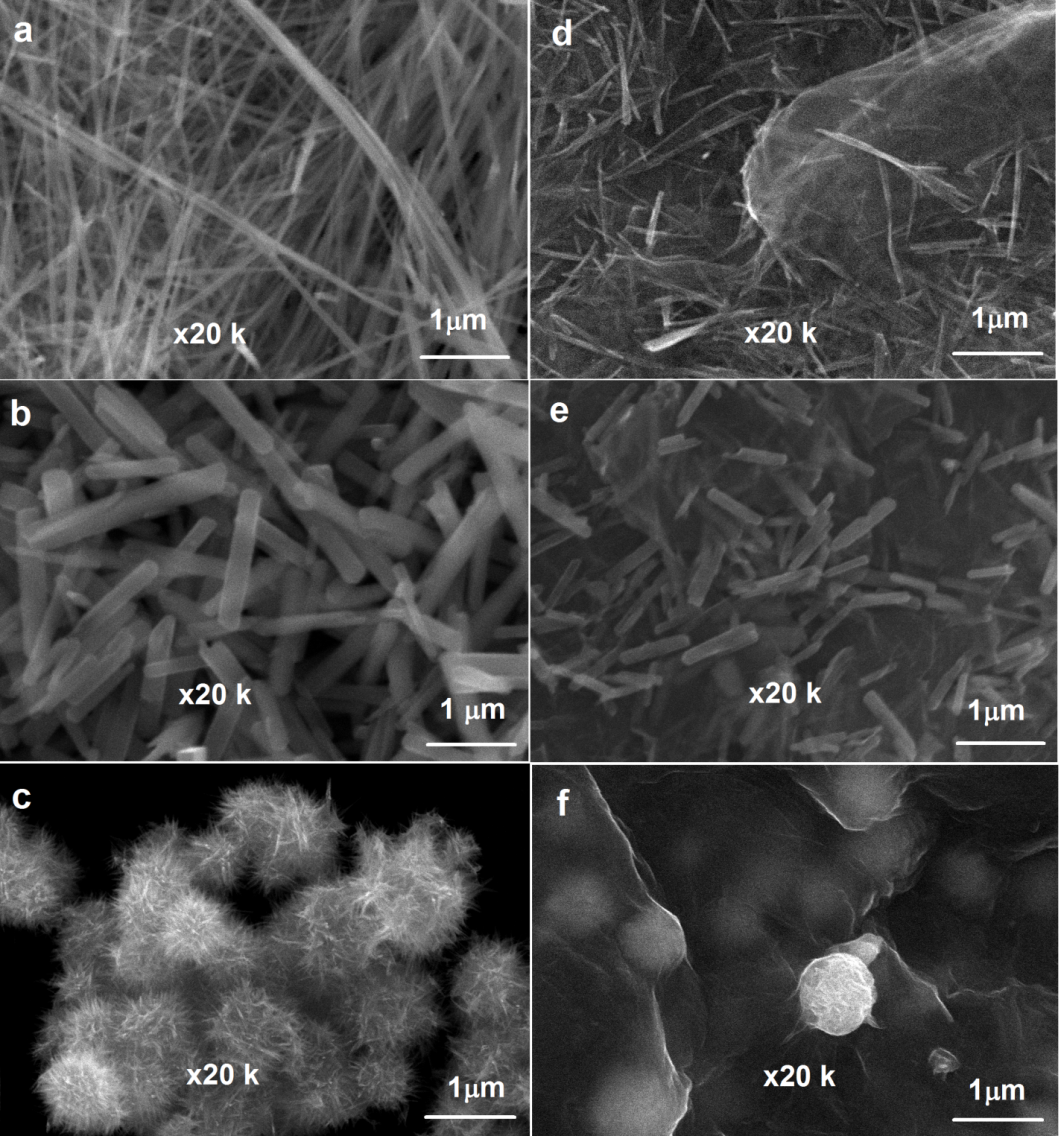
Surface morphology of (a) α-MnO_2_, (b) β-MnO_2_, (c) γ-MnO_2_, (d) graphene/α-MnO_2_, (e) graphene/β-MnO_2_, and (f) graphene/γ-MnO_2_ freestanding cathodes.

**Figure 2:**
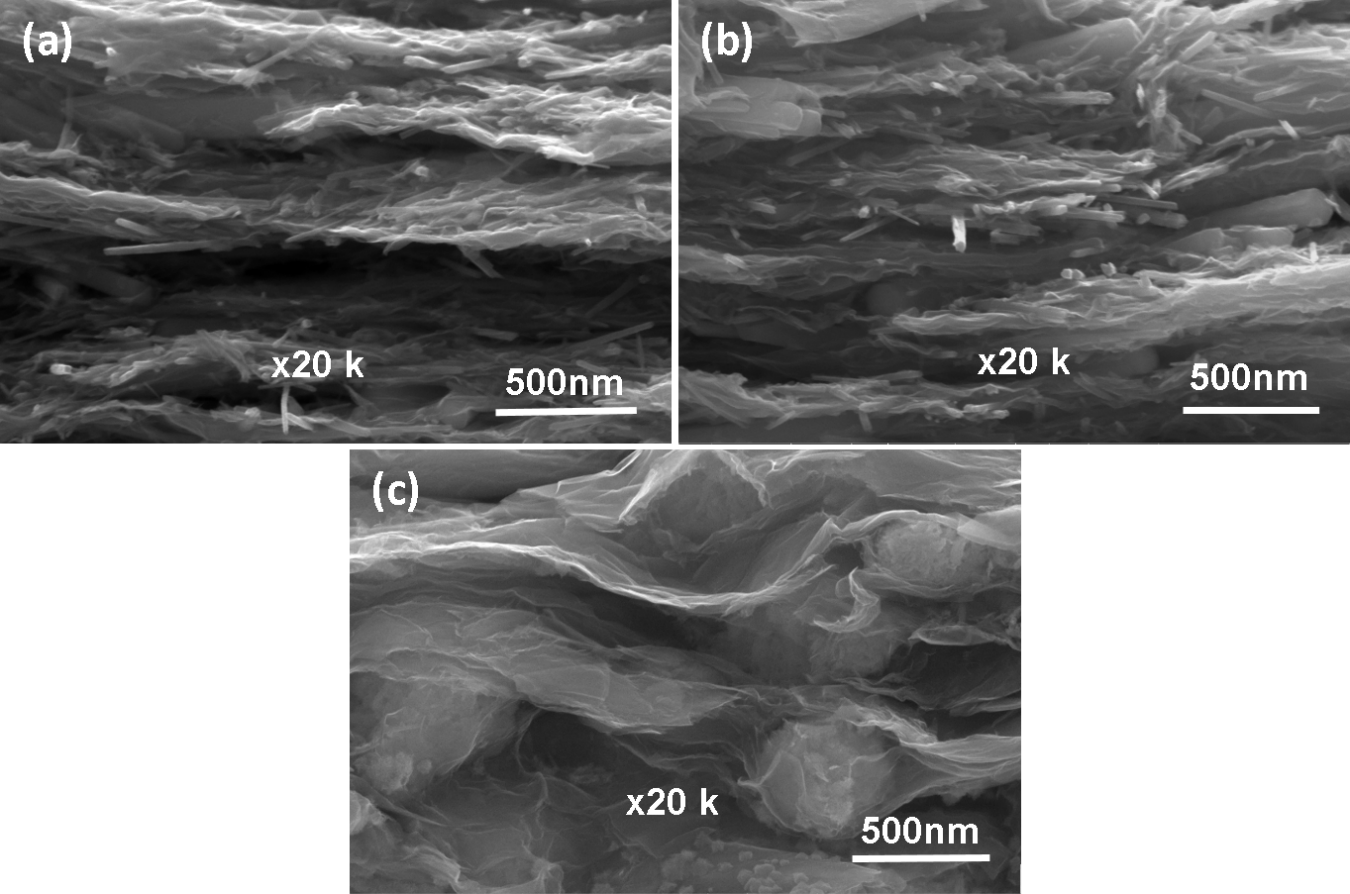
Cross-sectional SEM images of (a) graphene/α-MnO_2_, (b) graphene/β-MnO_2_, and (c) graphene/γ-MnO2 freestanding cathodes.

The surface morphologies of α-, β-, and γ-polymorphs of MnO_2_ and as-prepared graphene/MnO_2_ samples were investigated by SEM analysis. Figure 1a shows that the α-MnO_2_ nanostructure composed of uniform nanowires have 1–2 μm length and 40–60 nm average diameter. β-MnO_2_ (Figure 1b) shows that the as-prepared β-MnO_2_ sample has a nanorod structure with 0.5–1 μm length and 20–40 nm average diameter. The γ-MnO_2_ (Figure 1c) exhibits an urchin-like structure with 0.5–1 μm average diameter with very thin nanoneedles. The structure of graphene/MnO_2_ nanocomposites was also investigated. It can be seen from Figure 1d,e that α-MnO_2_ nanowires and β-MnO_2_ nanorods were homogeneously distributed on the surface and between the layers of graphene. Moreover, it also indicates that the urchin-like γ-MnO_2_ microspheres were wrapped by transparent graphene layers. In order to illustrate the dispersion of MnO_2_ polymorphs (i.e. not only the surface of graphene sheets, but also interlayers of graphene), cross-sectional characterization of graphene/MnO_2_ composite layers was implemented using SEM. As it can be seen from Figure 2, α-MnO_2_, β-MnO_2_ and γ-MnO_2_ structures were homogenously distributed and fixed between graphene layers.

**Figure 3:**
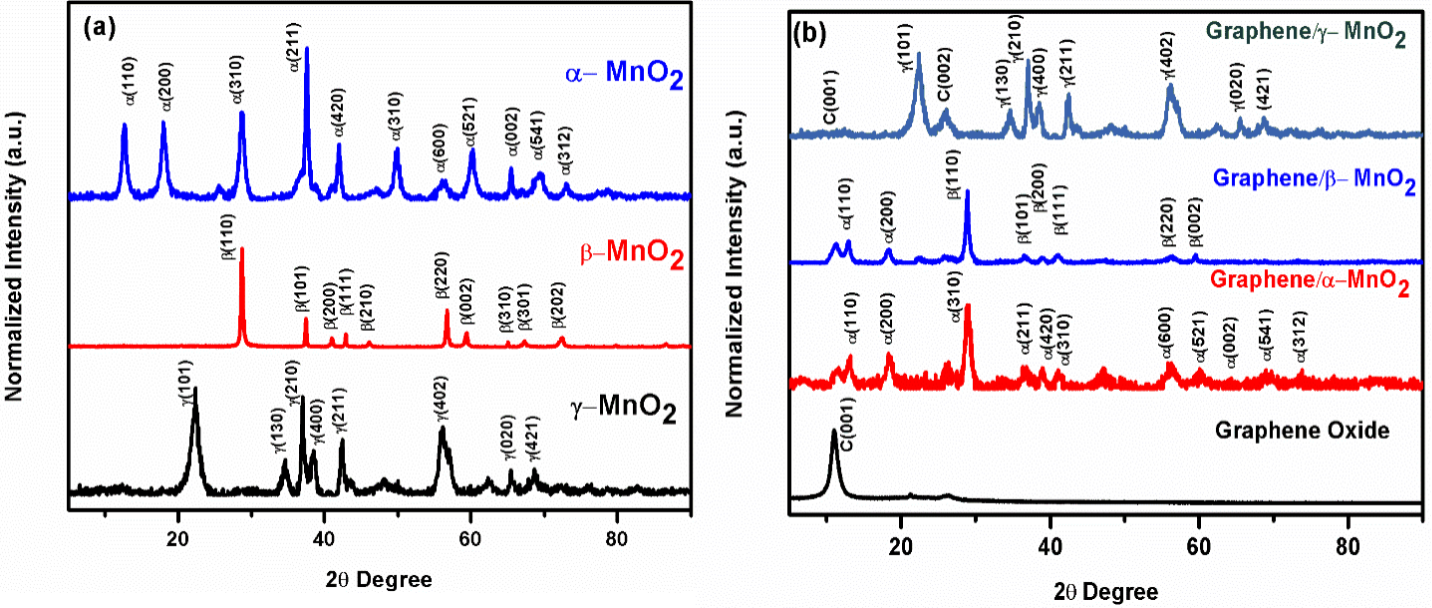
XRD patterns of (a) α-MnO_2_, β-MnO_2_, γ-MnO_2_, (b) graphene/α-MnO_2_, graphene/β-MnO_2_, and graphene/γ-MnO_2_ freestanding cathodes.

Figure 3a shows the XRD patterns of α-, β-, and γ-MnO_2_. The typical reflection peaks of α-MnO_2_ are observed at 2θ values of 12.7°, 18.0°, 28.6°, 36.7°, 38.6°, 41.9°, 49.7^o^, 56.4° 60.2°, 65.4°, 69.6°, and 72.9° corresponding to (110), (200), (310), (400), (211), (420), (301), (600), (521), (002), (541), and (312) planes of α-MnO_2_ crystals [20–21]. For β-MnO_2_, reflection peaks were observed at 2θ values of 28.7^o^, 37.4^o^, 41.0^o^, 42.9^o^, 46.1^o^, 56.7^o^, 59.4^o^, 65.0^o^, 66.8^o^, 67.3^o^, 72.3^o^, 79.7^o^ and 86.6^o^ corresponding to (110), (101), (200), (111), (210), (211), (220), (002), (310), (301), (202) and (321) planes of β-MnO_2_ [22]. Lastly, for γ-MnO_2_, reflection peaks were observed at 2θ values of 22.0°, 34.8°, 37.0°, 38.5°, 42.2°, 57.0°, 65.4° and 68.6°, corresponding to (101), (130), (210), (400), (211), (402), (020), (421) planes of γ-MnO_2_ [23]. Figure 3b shows XRD patterns of graphene oxide, graphene/α-MnO_2_, graphene/β-MnO_2_ and graphene/γ-MnO_2_ composite structures. The graphene peak observed at a 2θ value of 25.8^o^ indicates the (002) plane of carbon. However, there are still some remaining graphene oxide phases observed at 2θ values of 10.9^o^ in graphene/α-MnO_2_ and graphene/β-MnO_2_, while almost all graphene oxide is transformed to graphene in the graphene/γ-MnO_2_ structure [24–26].

**Figure 4:**
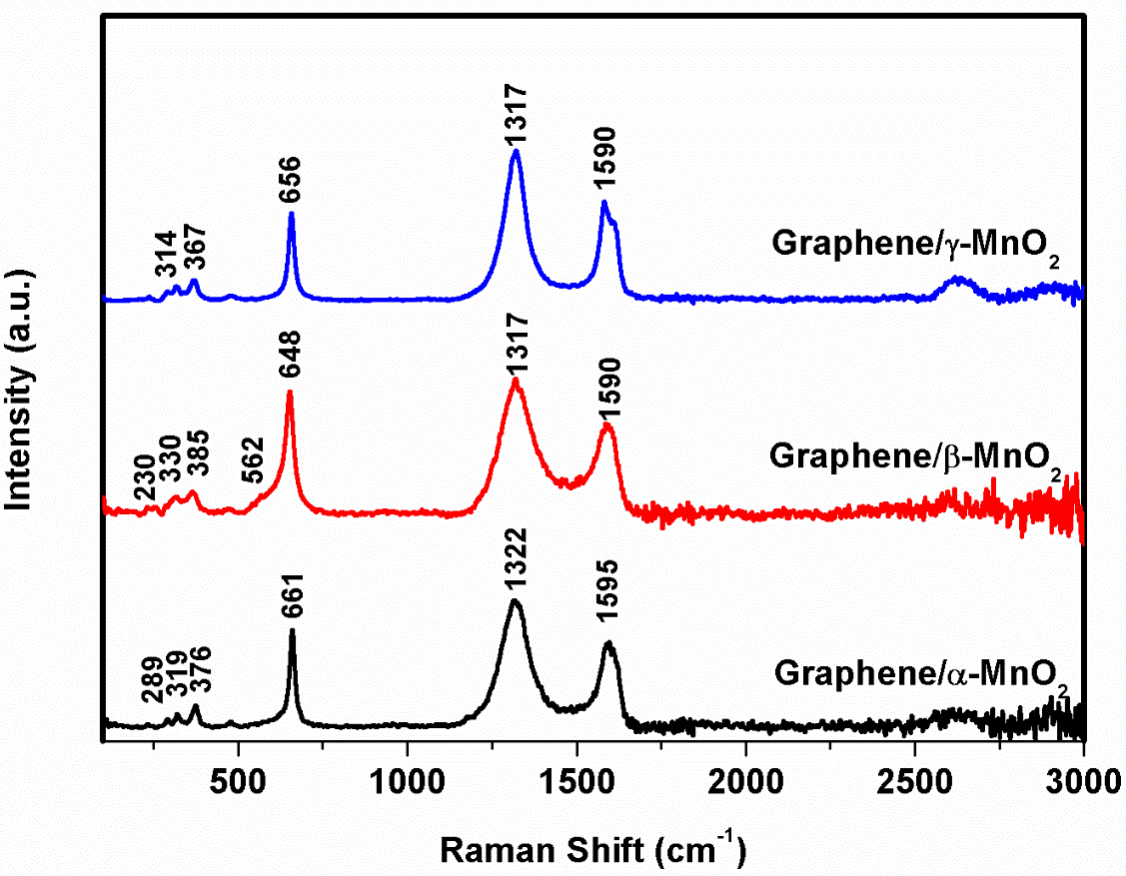
Raman spectra of graphene/α-MnO_2_, graphene/β-MnO_2_, and graphene/γ-MnO_2_ freestanding cathodes.

Further phase characterization of graphene/α-MnO_2_, graphene/β-MnO_2_ and graphene/γ-MnO_2_ composites was performed via Raman spectroscopy using a 785 nm laser and the results are shown in Figure 4. Although the Raman spectrum of MnO_2_ is generally used to characterize MnO_2_ structures, MnO_2_ structures may show different characteristic peaks due to different laser wavelengths and energy. Generally, in the Raman spectra of MnO_2_, the peaks between 500 and 700 cm^−1^ are attributed to the stretching mode of MnO_6_ octahedra [27] and the weak peaks between 200 and 400 cm^−1^ originate from the formation of Mn_2_O_3_ or Mn_3_O_4_ and correspond to the bending mode of O–Mn–O [28]. In the graphene/α-MnO_2_ composite, α-MnO_2_ shows three weak peaks at 289 cm^−1^, 319 cm^−1^ and 376 cm^−1^ and one strong peak observed at 661 cm^−1^. In graphene/β-MnO_2_ composites, three weak peaks at 230, 330 and 385 cm^−1^ and two strong peaks at 562 and 648 cm^−1^ are observed. Graphene/β-MnO_2_ exhibited two weak peaks at 314 and 367 cm^−1^ and one strong peak at 658 cm^−1^. The observed peaks at around 1320 and 1590 cm^−1^ are related to the D- and G-bands of graphene [29] in the graphene/MnO_2_ composite structures.

**Figure 5:**
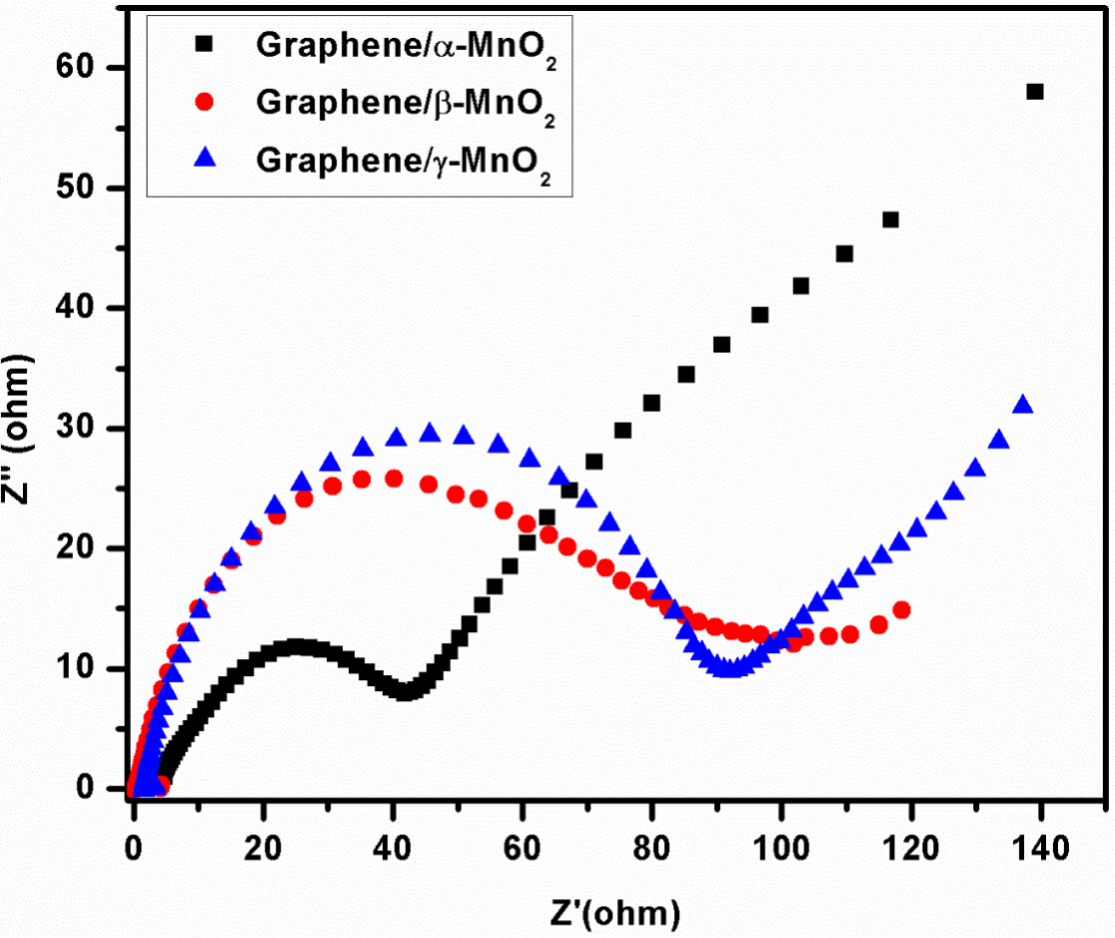
Nyquist curves of graphene/α-MnO_2_, graphene/β-MnO_2_, and graphene/γ-MnO_2_ freestanding cathodes.

In order to investigate the effect of different crystal structures of MnO_2_ in the graphene/MnO_2_ composites on the resistance of the cell, electrochemical impedance spectroscopy (EIS) measurements were performed and results are shown in Figure 5. The width of the Nyquist curves indicates the charge transfer resistance (*R*_ct_) of the graphene/α-MnO_2_, graphene/β-MnO_2_ and graphene/γ-MnO_2_ cathodes [30]. As seen from Figure 5, the graphene/β-MnO_2_ composite cathode has the largest width, showing *R*_ct_ = 102 Ω. Graphene/α-MnO_2_ with a *R*_ct_ = 42 Ω has a smaller width than that of graphene/γ-MnO_2_ with *R*_ct_ = 90 Ω. These *R*_ct_ values indicate that the graphene/α-MnO_2_ composite cathode has better electronic contact and conductivity among the produced freestanding graphene/MnO_2_ cathodes [31].

**Figure 6:**
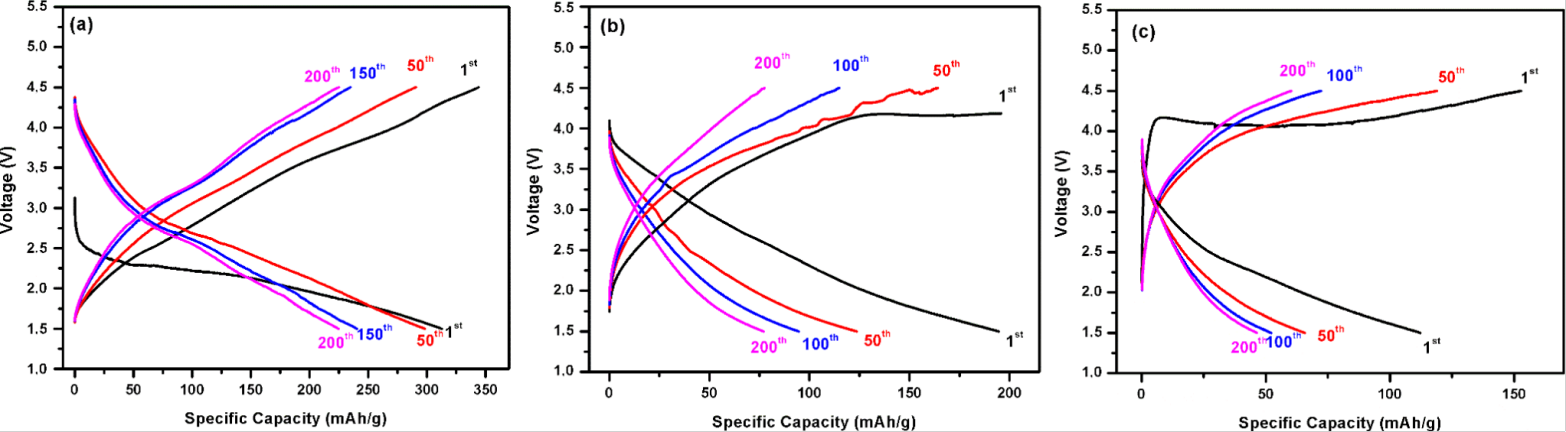
Galvanostatic charge/discharge profiles of freestanding (a) graphene/α- MnO_2_, (b) graphene/β-MnO_2_, and (c) graphene/γ-MnO_2_ cathodes.

The electrochemical performance of the as-synthesized cathodes was first evaluated by galvanostatic charge/discharge cycling at a constant current density of 254 mA g^−1^ in a voltage range from 1.5 to 4.5 V. In Figure 6, the typical charge/discharge profiles of freestanding graphene/α-MnO_2_, graphene/β-MnO_2_ and graphene/γ-MnO_2_ cathodes are given for the 1st, 50th, 100th and 200th cycles. As shown in Figure 6a, the graphene-supported α-MnO_2_ cathode exhibited a specific capacity of 321 mAhg^−1^ upon first discharge with an open-circuit potential of about 3.2 V and an average voltage of approximately 2.25 V. It can also be seen that the capacity of the graphene/α-MnO_2_ cathode was sustained with a small amount of capacity loss. This could be attributed to the wire-like structure of α-MnO_2_ allowing ions to pass from the cathode. When the graphene-supported β-MnO_2_ cathode was investigated (Figure 6b), it can be seen that the capacity was found to be much lower than for graphene/α-MnO_2_. While the first discharge capacity of graphene/β-MnO_2_ cathode was 198 mAhg^−1^, the graphene/γ-MnO_2_ cathode displayed a specific discharge capacity of 251 mAhg^−1^ (Figure 6c). The specific capacity of both graphene-reinforced β-MnO_2_ and γ-MnO_2_ electrodes decreased dramatically with increasing number of cycles. This could be attributed to the poor electrical conductivity and the textural modification during Li^+^ intercalation and de-intercalation processes. Cheng et. al. [32] prepared α-MnO_2_ cathodes and demonstrated a discharge capacity of 204.4 mAhg^−1^ for the first discharge with a constant current of 50 mAg^−1^. In our work, the as-prepared α-MnO_2_/graphene cathode was reached a specific capacity of 318 mAhg^−1^. This is probably due to graphene reinforcement, which increases the conductivity and electrochemical reaction of α-MnO_2_ with Li ions, as is reported in previous studies [10,13].

**Figure 7:**
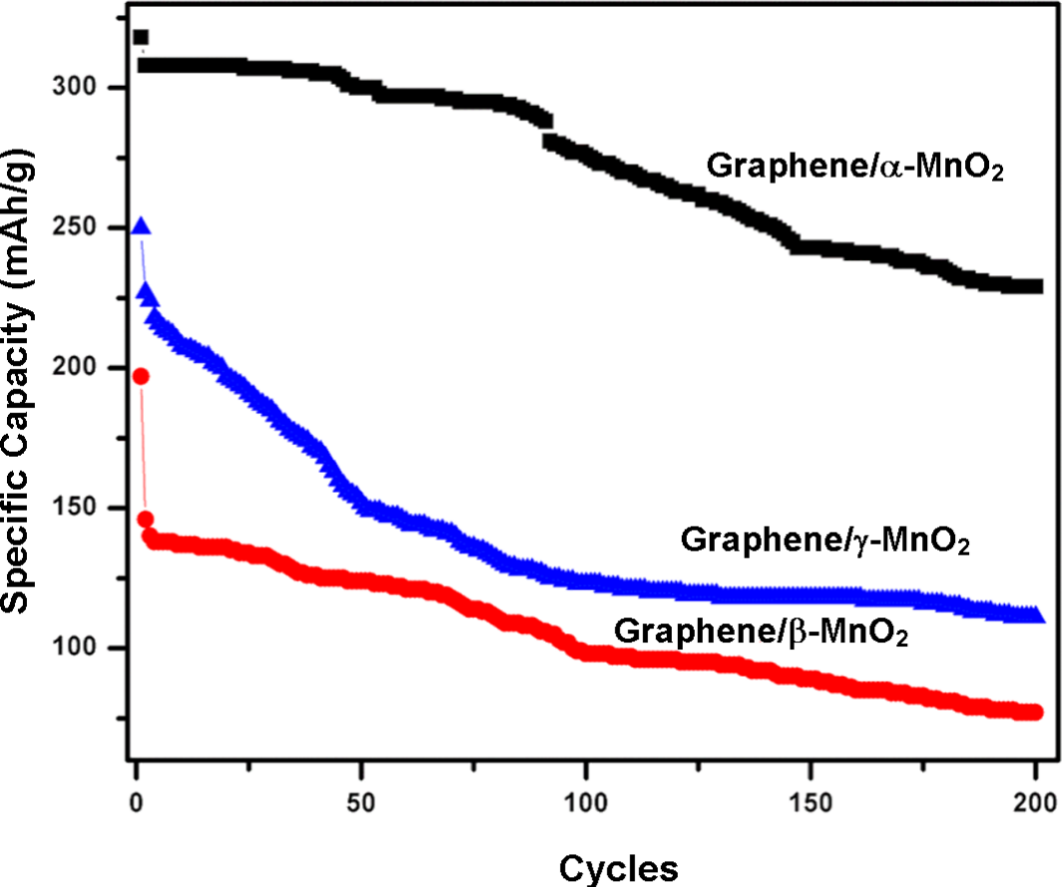
Electrochemical cycling tests of graphene/α-MnO_2_, graphene/β-MnO_2_, and graphene/γ-MnO_2_ freestanding cathodes.

Figure 7 reveals the cycling stability of graphene/α-MnO_2_, graphene/β-MnO_2_, and graphene/γ-MnO_2_ cathodes. A remarkable result is obtained from the graphene/α-MnO_2_ cathode which has an initial capacity of 321 mAhg^−1^. It can be seen that there is no sudden loss of capacity and between cycles 2 and 44 it exhibits almost a stable capacity of 305 mAhg^−1^. The total capacity loss is 27% during 200 cycles. Graphene/β-MnO_2_ and graphene/γ-MnO_2_ cathodes were also cycled until the 200th cycle but they exhibited very poor capacity retention when compared with the graphene/α-MnO_2_ cathode. Although both of these cathodes display a high capacity during the first cycle, the capacity value decreases dramatically during the second cycle. While the total capacity loss for the graphene/β-MnO_2_ cathode was 61%, the graphene/γ-MnO_2_ cathode showed a 55% capacity loss after 200 cycles. Tu et al. [33] also reported nanorods-shaped MnO_2_-graphene cathodes and a γ-MnO_2_ cathode, and they observed huge capacity reduction due to the formation of Li_2_MnO_3_.

## Conclusion

A facile and rapid microwave-assisted hydrothermal method was demonstrated to synthesize α-, β-, and γ-MnO_2_ phases. Freestanding graphene/MnO_2_ was successfully prepared with no further additives. The prepared nanocomposite samples were operated as positive electrodes for Li-ion batteries. The SEM images showed that α-MnO_2_ nanowires and β-MnO_2_ nanorods were homogenously dispersed not only at the surface, but also in the interlayer space of grapheme layers. Moreover, urchin-like γ-MnO_2_ microspheres were found wrapped by graphene nanosheets. The electrochemical cycling results demonstrated that the graphene/α-MnO_2_ cathode showed the best electrochemical performance among all prepared samples with an achieved initial capacity of 321 mAhg^−1^ and maintained its remarkable performance after many cycles. This study proved that α-MnO_2_ nanowires with graphene reinforcement could be promising cathodes for Li-ion batteries due to the high capacity and long cycle life.
